# Remote crisis intervention and suicide risk management in COVID-19 frontline healthcare workers

**DOI:** 10.3389/fpsyg.2023.1253179

**Published:** 2023-10-31

**Authors:** Rebeca Robles, Sara Infante, Miriam Feria, Iván Arango, Elsa Tirado, Andrés Rodríguez-Delgado, Edgar Miranda, Ana Fresán, Claudia Becerra, Raul Escamilla, Eduardo Angel Madrigal de León

**Affiliations:** ^1^Global Mental Health Research Center, Ramón de la Fuente Muñiz National Institute of Psychiatry, Mexico City, Mexico; ^2^Directorate of Clinical Services, Ramón de la Fuente Muñiz National Institute of Psychiatry, Mexico City, Mexico; ^3^Laboratory of Clinical Epidemiology, Subdirectorate of Clinical Research, Ramón de la Fuente Muñiz National Institute of Psychiatry, Mexico City, Mexico; ^4^General Directorate Ramón de la Fuente Muñiz National Institute of Psychiatry, Mexico City, Mexico

**Keywords:** crisis intervention, suicide, healthcare workers, COVID-19, e-mental health, implementation science

## Abstract

**Introduction:**

Despite the propagation of virtual mental health services for vulnerable groups during COVID-19 pandemic, the implementation and evaluation of remote evidence-based practices (EBP) to manage them in low- and middle-income countries remains scarce. In the current study, we describe and evaluate the implementation process and clinical impact of brief, remote, manualized EBP for crisis intervention and suicide risk management among healthcare workers attending patients with COVID-19 (COVID-19-HCWs) in Mexico.

**Methods:**

The implementation process comprised community engagement of volunteer mental health specialists, creation of new clinical teams with different disciplines and skills, intervention systematization through manuals and education through 4-h remote training as main strategies. Mexican COVID-19-HCWs who had used a free 24-h helpline rated their pre- and post-intervention emotional distress. Therapists recorded patients’ pre-intervention diagnosis, severity, and suicide risk, the techniques used in each case, and their post-treatment perception of COVID-19-HCWs’ improvement at the end of the intervention.

**Results:**

All techniques included in the intervention manual were employed at least in one case (*n* = 51). At the beginning of the intervention, 65.9% of the COVID-19-HCWs were considered moderately ill or worse according to Clinical Global Impression-Severity (CGI-S) scores, whereas at the end, 79.4% of them were perceived as much or very much improved according to CGI-Improvement scores (CGI-I), and their emotional distress had been significantly reduced (*p* < 0.001).

**Discussion:**

This prospective study provides evidence that implementation of remote EBP is feasible and useful to reduce emotional distress and suicide risk among COVID-19-HCWs from a middle-income country. However, this study was limited by lack of a control group, improvement ratings provided by therapists and non-anonymous satisfaction ratings.

## Introduction

Healthcare workers attending patients with COVID-19 (COVID-19-HCWs) constituted one of the known vulnerable groups in terms of mental health problems (MHP) and suicide risk (Adhanom, 2020). Thus, in parallel with universal suicide prevention strategies designed for the entire population (Mann and Currier, 2011), selective interventions for this (and other vulnerable groups) had to be provided, including mental disorders and suicide risk assessment (Gunnell et al., 2020) as well as indicated evidence-based practices (EBP) targeting those experiencing emotional crises and suicidal risk.

Main examples of EPB for these purposes include psychological first aid (American Psychiatric Association, 1954; Vernberg et al., 2008; Corey et al., 2021), pharmacotherapy for mental disorders (Brent, 2016), brief cognitive-behavioral therapy (CBT) (DeCou et al., 2019), and safety planning (Stanley and Brown, 2012). which had to be adapted to ensure their safe implementation in the context of such highly contagious virus by increasing the development and use of helplines, telemedicine and other digital methods (Gunnell et al., 2020).

The propagation of remote mental health services during COVID-19 might expand access to mental health care during and beyond the pandemic (Wind et al., 2020; Zhou et al., 2020), which is unfortunately low among healthcare students and professionals, even among those with substantial risk factors for suicide (Givens and Tjia, 2002). Barriers to using face-to-face mental health services, which are often offered in institutions where healthcare students and professionals work, include lack of time and fear of documentation in academic or professional records. Such barriers might be solved through remote interventions (e.g., hotlines) not associated with these institutions.

Scientific evidence demonstrates that EBP have effectively been applied remotely to reduce distress and suicide risk in general population and in HCWs (Berrouiguet et al., 2018; Pospos et al., 2018; Reinhardt et al., 2019). According to a systematic review of studies carried out prior to the pandemic, Pospos et al. (2018), showed that web-based tools and mobile applications that incorporate techniques usually employed as part of psychological first aid and suicide prevention (e.g., deep breathing, cognitive-behavioral techniques to cope with unpleasant thoughts and emotions, development of a customized safety plan and grounding exercises), are useful to improve healthcare student’s and professional’s coping, thus mitigating psychological distress and suicide risk.

However, it is still necessary to increase research efforts on which methods are useful for achieving their effective implementation (adoption by clinicians) in the contexts in which they are applied (Wasserman et al., 2020) when significant changes in clinicians’ routine delivery methods (remote vs. face-to-face), and patients (HCW-COVID-19) are in place.

In line with this, according to the emerging field of implementation science of EBP—as opposed to the sciences for their development through clinical trials—, research in this field should be oriented toward understanding the methods for promoting their incorporation into clinical practice (Berrouiguet et al., 2018). Therefore, implementation studies typically focus on the impact of specific strategies on the rates and quality of use of EBP, the proportion of patients who attend a minimum number of treatment sessions, and the adaptations required to improve the implementation process.

The present study sets forth the results of the evaluation of the implementation process and clinical impact of remote EBP designed to address emotional crises and suicide risk in Mexican COVID-19-HCWs, as part of a country-level strategy coordinated by the Mexican Ministry of Health (through its National Institute of Psychiatry), in collaboration with the National Autonomous University of Mexico (UNAM) since the beginning of the COVID-19 pandemic in the country.

Specific objectives include describing: (1) COVID-19-HCWs’ help-seeking process (including their sociodemographic and professional profile, sources of referral and reasons for consultation), (2) COVID-19-HCWs’ MHP and suicide risk according to service providers, (3) the EBP most frequently used by providers (and the reasons for adopting them), (4) the clinical utility of the intervention according to providers (by comparing their perception of the severity of COVID-19-HCWs’ MHP at the start of the intervention with COVID-19-HCWs’ improvement at the end of it), and (5) the acceptability and clinical utility of the intervention according to users (by comparing COVID-19-HCWs’ pre-post perception of emotional distress).

## Materials and methods

### Participants: clinicians, and patients

A total of 18 clinicians voluntarily participated; 5 were psychiatrists attending cases with suicide risk and/or in need for pharmacotherapy in addition to psychological techniques; and 13 were psychologists with at least a master’s degree. The sample of patients comprised 51 Mexican COVID-19-HCWs over 18 years old who voluntarily agreed to participate in the study, be assessed by a mental health specialist before and after the intervention, and attended at least one intervention session, from April 17 to December 15, 2020, the period encompassing the clusters of cases scenario (when a country experiences cases clusters in time, geographic location, or common exposure; World Health Organization, 2020) and the community transmission scenario (when a country experiences larger outbreaks of local transmission; World Health Organization, 2020), including the first COVID-19 peak in Mexico.

### Variables and measures

*Sociodemographic, professional, and COVID-19-related variables* were evaluated using self-report questions on subjects’ sex, age, marital status, education, profession, personal COVID-19 status, and the COVID-19 status of friends and relatives.

#### Contact, reason for consultation, and emotional distress

Each subject was asked how they had found out about the free 24-h helpline and the reason for their call. The answers to these open-ended questions were recorded in a previously designed format for patients seeking psychological crisis intervention and suicide risk management. They were subsequently coded for analysis. Emotional distress was assessed at the beginning and end of the intervention through the following sentence: *“Which number best describes how much emotional distress you are experiencing right now, on a scale of 0 to 10?”* (Almanza-Muñoz et al., 2008).

#### Diagnostic impression and overall clinical impression

Each mental health professional recorded their diagnostic impression and assessed the severity of the symptoms reported during the psychological crisis using the Clinical Global Impression (CGI)-Severity Scale (CGI-S), rated on a 7-point Likert scale ranging from 1 (normal) to 7 (among the most severely ill patients). At the end of the intervention, the CGI-Improvement Scale (CGI-I) was assessed. CGI-I scores range from 1 (very much improved) to 7 (very much worse) (Guy, 1976).

#### Suicide risk assessment

The level of suicide risk (mild, moderate, high) was assessed in keeping with the risk of suicide module of the Mini-International Neuropsychiatric Interview (MINI) (Sheehan et al., 1997; Ferrando et al., 1998), comprising six items with a yes/no answer. In addition, the type of intervention required was determined by the total number of the ten risk factors included in the SAD Persons scale (0–4 = ambulatory treatment, 5–19 = hospitalization) (Patterson et al., 1983; Comité de Consenso de Catalunya en Terapéutica de los Trastornos Mentales, 2005; Gobierno de Canarias, 2008).

#### Clinicians’ adoption of EBP

To determine the level of adoption of the manual and the need to incorporate new techniques, clinicians were asked to record the techniques used and the reasons for their use in every session and case in an *ad hoc* form designed for the study.

*Acceptability of the Intervention* was evaluated using three questions; two regarding the level of patients´ satisfaction with the contents and intervention modality (remote), and the degree of complexity of the intervention, to be answered on a 5-point Likert scale, where 0 = not at all satisfied/complex, 1 = not very satisfied/complex, 2 = moderately satisfied/complex, 3 = very satisfied/complex, and 4 = fully satisfied/complex. At the end, a dichotomous question (yes/no) was included on whether subjects would recommend the intervention to other health workers coping with COVID-19.

### Procedures

#### Main implementation strategies and outcomes

Implementation strategies included *community engagement* (Pinto et al., 2021) of volunteer mental health specialists willing to attend COVID-19-HCWs (psychiatrists and psychologists from de National Institute of Psychiatry with extensive experience treating emotional crises and/or psychiatric urgencies), in order to warrantee the availability of sufficient professional resources and the *creation of new clinical teams* with different disciplines and skills (Powell et al., 2015); *intervention systematization* through manuals and *education* through 4-h. online training session (Powell et al., 2015) in order to strength their skills to implement remote EBP to address emotional crises and suicide risk; and allow better assessment of implementation and clinical outcomes (Pinto, 2013).

Training session’s topics, teaching strategies and time employed to each topic were: 1) Use of the intervention manuals (described below) through conference and group discussion on the application of each strategy to case vignettes (of HCWs in emotional crises and/or with suicide risk during the pandemic) during two hours, 2) Process for referring patients to a psychiatrist, different virtual clinics and services if needed, though conference and exemplification during 30 min, and 3) Administration of the instruments and recording the data for the study (including the additional techniques they considered necessary for each case), through conference, groups exercises applying instruments to case vignettes and recording data in corresponding Excel formats, during 90 min. At the end of the online training session on intervention and evaluation methods, a case vignette was presented to determine interrater reliability between clinicians in the assessment of the patients´ initial severity, suicide risk and improvement after intervention. The Fleiss’ kappa coefficient was determined with values over 0.80 obtained in all cases, reflecting an adequate level of agreement between clinicians.

The main expected implementation outcome was a high level of adoption of EBPs by clinicians. Secondly, we expected high patient satisfaction and significant clinical utility of the intervention. Thus, to be part of the study’s sample, the record of the techniques employed during intervention sessions must be totally complete, and the evaluations of patients´ clinical improvement and treatment satisfaction were not mandatory.

#### Remote recruitment, evaluation, and intervention

Mexican COVID-19-HCWs were invited to use the free 24-h helpline to cope with emotional crises and/or suicidal thoughts through several sources, including a brief online evaluation and referral to treatment tool (at the national COVID-19 website: coronavirus.gob.mx), which automatically delivered personal feedback after completion of valid, reliable scales, including specific contact information on virtual clinics and other specialized services, if required, (2) the coordinators or therapists of other national virtual clinics for COVID-19-HCWs, (3) the National Institute of Psychiatry’s website and social networks, (4) press conferences and remote meetings with COVID-19-HCWs and COVID-19 health care center authorities (mainly at congresses and academic sessions), and (5) posters at the entrance to the COVID-19 zones of COVID-19 health care centers.

The free 24-h helpline for managing emotional crises and/or suicidal thoughts was answered by one of the participating clinicians. All of them were equipped with a mobile phone to answer the helpline according to a scheduled agenda of days and hours to be covered by each one to cover the service 24 h. Then, clinicians and patients decided together the best way to communicate remotely, whether it was the phone or a session using a virtual platform (for example, Zoom).

Sociodemographic, professional, COVID-19-related variables, source of referral, reasons for consultation and emotional distress were registered at the beginning of the intervention, prioritizing the attention of the patients. At the end of first session, clinicians registered their diagnostic and overall clinical impression, as well as patients´ suicide risk.

Crisis intervention was delivered according to the operational manual for remote psychosocial care during the COVID-19 pandemic in Mexico (Álvarez et al., 2020), which include a detailed description of a psychological intervention in emergencies based on Crisis Intervention Model and Cognitive Behavioral Therapy. Table 1 includes a description of the content and adaptations for COVID-19 pandemic in each manual. Suicide risk was handled using Dialectic Behavioral Therapy techniques (such as TIPP and mindfulness skills), according Linehan’s (2014) DBT Skills training manual.

**Table 1:** Manual for crisis intervention (Álvarez et al., 2020): content and adaptations for COVID-19 pandemic.

Themes and subthemes	Adaptations for COVID-19 pandemic
*Emergencies and crisis*	
Definition and types of emergencies and crisis. Psychological reactions: Acute stress reaction, posttraumatic stress disorder, depression, and generalized anxiety disorder.	Inclusion of health emergencies, highlighting specific stressors of COVID-19 pandemic (such as quarantine, physical/social distance, fear of contagion)
*Psychological first Aid*	
General principles and objectives. Therapeutic alliance, active listening and containment, encouraging emotional expression and validating emotions, problem solving and positive reinforcement, identification and management of suicide risk (including reference to psychiatric evaluation if needed)	Emphasis on online implementation and teamwork (as part of the health emergency personnel). Use of the brief online evaluation and referral to treatment tool for COVID-19 HCWs (described in methods section) and other helplines. Inclusion of the importance of psychoeducation on dealing with grief during COVID-19.
*Crisis Intervention*	
Definition and guidelines to stablish a safety and crisis plan including personal, social and institutional resources. Deactivation techniques: deep breathing, relaxation (imagery and muscular), meditation and mindfulness. Physiological activation: body self-massage, body movement. Thought modification: ABC model, weighing the evidence and generating alternative interpretations, reattribution, semantic method, terms definition, survey method, and cost–benefit analysis.	Consideration of need for pharmacological and/or other specialized treatments and use of the brief online evaluation and referral to treatment tool for COVID-19 HCWs (described in methods section). Inclusion of specific examples expressing cognitive distortions during COVID-19 pandemic. Inclusion of case vignettes (e,g, a women with extreme worries about COVID-19 contagion due to one simple symptom, and the recommended intervention.
*Healthcare workers self-care* Self-care measures (nutrition, rest, exercise, social contact, avoiding maladaptive strategies (e.g., use of alcohol in the face of negative emotions)	Inclusion of recommendations for health care professionals during COVID-19

However, therapists could use the techniques of their choice for each case, regardless of whether they were included in the intervention manuals, including pharmacotherapy for MHP, and hospitalization for severe cases. At the end of each session, clinicians recorded the techniques used and the reasons for their use, as well as new important information for case management if they considered it necessary (e.g., antecedents of mental health problems and psychological/pharmacological treatments, comorbidity, etc.). At the end of the intervention, clinicians registered patients´ global improvement and patients´ acceptability of the intervention.

### Data analyses

All analyses were performed using SPSS-X v.21.0. All descriptive information was determined by frequencies and percentages for categorical variables and means and standard deviation for continuous variables.

Demographics, professional, COVID-19-related variables, reason for consultation (including suicide risk), diagnostic impression and CGI scores among HCWs were described as well as the frequency and percentage of sessions and the use of the techniques included in the intervention manual and the additional ones. We also recorded subjects’ degree of satisfaction with the contents and modality of the intervention, and perception of its complexity.

Emotional distress before and after the intervention (in those who finished the intervention and completed both evaluations) was compared using repeated-measures Student’s t-tests with a prefixed alpha value of *p* < 0.05. Cohen’s d for t-tests were obtained to determine the effect sizes of the comparisons.

Finally, a content analysis was conducted by categorizing the meanings (Kvale, 1996) of the reasons for the use of additional techniques, to reveal the reason for their use by the therapist.

## Results

A total of 234 HCWs was attended in the virtual clinic for crisis intervention and suicide risk management during the period of the study. 183 HCWs were excluded from the study’s sample given the reports of their evaluations were not delivered by the treating psychotherapist given they did not have time to complete the records (in 150 cases) or patients did not accept to complete the evaluations for the study (n = 33). Reports of the remaining 51 HCWs were provided for the present study: 66.7% (*n* = 34) was women and the remaining 33.3% (n = 17) men, with a mean age of 36.4 (S.D. = 8.5, range 21–60) years. All of them began treatment but three (5.9%) dropped out (two after the first session and one after the fourth session), meaning that 48 (94.1%) completed the intervention. Of the latter, 51.0% (*n* = 25) failed to complete the questionnaire on the acceptability of the intervention arguing they did not have time to do so. Table 2 shows the main demographic, professional and COVID-19-related variables of the sample. None of the participants was receiving other types of mental health care during the study.

**Table 2:** Demographic, professional and COVID-19-related variables (*n* = 51).

Variables	Categories	Descriptives
Sex, *n (%)*	Men	17 (33.3)
	Women	34 (66.7)
Age, *mean (S.D; range)*		36.4 (8.5; 21–60)
Marital status, *n (%)*	Partnered	22 (43.1)
	Unpartnered	29 (56.9)
Educational attainment, *n (%)*	Undergraduate	7 (13.7)
	Bachelor’s degree	27 (52.9)
	Specialty degree	9 (17.6)
	Master’s or PhD degree	8 (15.7)
Professional profile, *n (%)*	Medicine ^a^	20 (39.2)
	Psychology	9 (17.6)
	Nursing	8 (15.7)
	Social work	3 (5.9)
	Other	11 (21.6)
Primary institution, *n (%)*	Federal Ministry of Health (SSA)	21 (41.2)
	Public State Health Services	9 (17.6)
	Private practice	8 (15.7)
	Other	13 (25.5)
COVID-19 status, *n (%)*	No symptom	30 (58.8)
	Acute respiratory disease	8 (13.7)
	Confirmed COVID-19 diagnosis	13 (25.5)

a Includes undergraduate physicians, general practitioners, interns, medical specialty residents, and specialist physicians.

As can be seen in Table 3, the most frequent source of referral to the helpline was another HCW. The main reasons for consultation included having tested positive for COVID-19 (*n* = 10, 21.3%) or the suspicion or fear of having COVID-19 (n = 9, 19.1%), as well as experiencing the death of close friends or loved ones from COVID-19 (n = 10, 21.3%). According to the mental health professionals answering the helpline, more than 60 % of the sample presented some form of depression (with or without anxiety symptoms), while nearly 30 % were at risk of suicide.

**Table 3:** Source of referral to helpline, reason for consultation, diagnostic impression, and suicide risk.

Variables	Categories	*n* (%)
Source of referral to helpline (*n* = 47^a^)	Another HCW	16 (34.0)
	Official media	9 (19.1)
	National Autonomous University	8 (17.0)
	Social media	4 (8.5)
	Physical propaganda	2 (4.3)
	Coordinator of another clinic	1 (2.0)
	Other	7 (14.9)
HCWs’ reason for consultation (*n* = 47^b^)	COVID-19 positive test	10 (21.3)
	Death from COVID-19 (family, colleagues, patients)	10 (21.3)
	Fear of COVID-19 contagion	9 (19.1)
	Mental health problems (depression, anxiety, etc.)	6 (12.8)
	Having COVID-19 symptoms/waiting for test results	4 (8.6)
	Helping others with their mental health problems due to COVID-19	3 (6.3)
	Colleagues infected with COVID-19	2 (4.3)
	Relationship problems (friends/family)	1 (2.1)
	Fear of failing to complete studies due to the pandemic	1 (2.1)
	Death of family members from non-COVID-19 causes	1 (2.1)
Therapist’s diagnostic impression (*n* = 51)	Depression with anxiety symptoms	29 (56.9)
	Anxiety disorder	12 (23.5)
	Depression	5 (9.8)
	Alcohol dependence/abuse	2 (3.9)
	Panic disorder	1 (2.0)
	Health anxiety disorder	1 (2.0)
	Grief due to death of family member from COVID-19	1 (2.0)
Suicide risk (*n* = 51)	None	22 (43.1)
	Minimal risk, ambulatory treatment	7 (13.7)
	Moderate risk, ambulatory treatment	6 (11.8)
	High risk, hospitalization	2 (3.9)
	Therapist considered it unnecessary to assess subject	14 (27.5)

a 7.8% (*n* = 4) HCW failed to provide information on how they found out about the hotline.

b 7.8% (*n* = 4) HCW did not give their personal reasons for attending the service.

### Intervention techniques

The number of sessions and techniques used for the subjects was reported, including those who dropped out of treatment. The average number of intervention sessions was 3.5 (S.D. = 2.5, range = 1–12); initial sessions were longer than follow up sessions (60 to 90 min vs. 40 to 60 min). Table 4 presents the frequency of use of the intervention techniques, comprising those contained in the manual, as well as those not included in it yet considered necessary for the cases under treatment by the therapist. Table 4 also shows the reasons for the use of these additional techniques, which are classified into three main categories: 1) Psychopharmacology, 2) Self-disclosure and 3) Use of metaphors.

**Table 4:** Type, frequency of intervention techniques throughout sessions, reasons for use of additional techniques (*n* = 51), CGI and emotional distress assessment.

Manual techniques		Additional techniques	
	*n (%)*	*n* (%)	Reasons (examples)
Listen & containment Emotion validation Breathing & relaxation Thought modification Safety plan Crisis plan Meditation and mindfulness TIPP skills Problem solving: use of COVID-19 protective measures Positive reinforcement Self-care measures Reasons for living Psychoeducation and data on dealing with grief Problem solving: family issues Chain analysis of problem beahaviour (alcohol use) Problem solving: Assertiveness in the face of work pressure Problem solving: Establishing limits to over-involvement in work Assessment of support network Emotional expression (catharsis) Psychoeducation for depression Referral to addiction intervention	46 (90.2) 23 (45.1) 16 (31.4) 11 (21.6) 6 (11.8) 4 (7.8) 4 (7.8) 3 (5.9) 3 (5.9) 3 (5.9) 3 (5.9) 2 (3.9) 2 (3.9) 2 (3.9) 1 (2.0) 1 (2.0) 1 (2.0) 1 (2.0) 1 (2.0) 1 (2.0) 1 (2.0)	Psychopharmacology 20 (39.2) Self-disclosure 8 (15.7) Use of metaphors 2 (3.9)	*“Antecedents of depression four to five* ye*ars ago. Current persistent demotivation and lack of energy; anxiety related to returning to work and being re-infected. Suggest patient should continue with self-prescribed anxiolytic treatment and being taking an antidepressant.”* *“To be empathetic by showing that I understand the fear of contagion as part of the vulnerability of being medical personnel and then suggest focusing on the here and now to modify catastrophic thoughts that increase anxiety.”* *“The metaphor about catastrophic thoughts as if they were a runaway horse was used. Thought stopping techniques or other anxiety control tools are the reins that hold them back. The patient was asked to repeat to herself that these catastrophic thoughts are thoughts, not realities.”*

Percentages by column do not sum 100 given different techniques were employed in each case (for example: in any case “listen and contain” was the only strategy employed).

### Clinical utility of the intervention

The CGI assessment at the beginning and end of the intervention and the comparison of the emotional distress experienced are included in Table 5. As can be seen, at the beginning of the intervention, more than half of the HCWs were classified as moderately ill, whereas by the end of the intervention, 79.4% were considered “very much” or “much” improved by the treating mental health professional. Seven women (23.3% of the total female participants) were classified as moderately to severely ill, while all men were assessed with lower illness severity (borderline moderately ill, to moderately ill), which is in line with women’s initial report of higher distress than men. However, at the end of the intervention, no differences in the distress experienced by sex were founded. Congruently, both women and men reported a significant reduction in emotional distress after the intervention, with “large” effect sizes according to Cohen’s d coefficients (Cohen, 1992). Interestingly, higher proportion of men reported being significantly improved (according to CGI-I scale) at the end of the intervention.

**Table 5:** Severity of mental health condition at the start of the intervention, improvement at the end of the intervention and HCWs’ pre-post perception of emotional distress: data from the total sample and by sex.

CGI severity (n = 47)	Men (*n* = 17)	Women (*n* = 30)	Improvement (*n* = 34)	Men *n* (%)	Women *n* (%)	Distress	Men media (SD)	Women media (SD)
2 = borderline ill 3 (6.4) 3 = mildly ill 11(23.4) 4 = moderately ill 26 (51.0) 5 = markedly ill 4 (8.5) 6 = severely ill 3 (6.4)	3 (17.6) 3 (17.6) 11 (64.7) - -	- 8 (26.7) 15 (50.0) 4 (13.3) 3 (10.0)	1 = very much 22 (64.7) 2 = much 5 (14.7) 3 = minimally 5 (14.7) 4 = no change 2 (5.9)	9 (90.0) 1 (10.0) - -	13 (54.2) 4 (16.7) 5 (20.8) 2 (8.3)	Initial (n = 45): 7.3 (2.1) Final (n = 44): 2.4 (2.4) t = 12.1, *p* < 0.001 Cohen’s *d* = 2.3	6.1 (1.7) 1.6 (1.4) t = 8.1, *p* < 0.001 Cohen’s *d* = 2.8	8.0 (2.1) 2.8 (2.8) t = 12.1, *p* < 0.001 Cohen’s *d* = 2.1

Due to the empty cells on the CGI Severity and Improvement scales, no statistical comparisons between men and women were performed.

### Acceptability of the intervention

Of the 48 HCWs who completed the intervention, a subsample of 26 reported their level of satisfaction with and perception of the complexity of the intervention. All of them (100%) stated that they were “totally satisfied” with the contents of the intervention. Moreover, the majority (n = 17, 65.4% of the subsample) answered that the intervention was “not complex” (followed by 19.2% (n = 5) who considered it “not very complex” and only 7.7% (n = 2) reported that it was “very complex.” All the subjects reported that they would recommend it to their colleagues. Almost all the HCWs were “totally satisfied” (n = 24, 92.3% of the subsample) or “very satisfied (n = 2, 7.7%) with the remote modality of the intervention.

## Discussion

Present implementation study carried out a preliminary or basic evaluation of the utility of *community engagement* (Pinto et al., 2021), *creation of new clinical teams*, *intervention systematization* and *education* (Powell et al., 2015) as the implementation strategies to achieve clinicians´ adoption of remote EBP to address emotional crises and suicide risk, and therefore some indicators of significant clinical improvements and high levels of satisfaction with the intervention among a sample of COVID-19-HCWs in a middle income country (Mexico).

According to our results, these implementation strategies were useful to attain high rates of use of EBP, referred in implementation science as adoption level (Proctor et al., 2011). All techniques included in the intervention manual were employed at least in one of the 51 cases included in the study, particularly listening and providing containment (as a psychological first aid employed to help COVID-19-HCWs feel calm), which were adopted by clinicians to treat 90% of COVID-19-HCWs. This figure is in line with Buselli et al. (2020) previous report on the high perception (around 70%) of CBT techniques´ appropriates for the psychological care of Italian COVID-19-HCWs.

Moreover, the evaluation of the EBP´ level of adoption allows the exploration of specific techniques that are considered necessary to add by participating therapists (Proctor et al., 2011), which was expected given the need for psychological techniques to address patients in extraordinary stressful circumstances (Chen et al., 2020). In general terms, the manualized intervention might be improved by the addition of EBP considered relevant for this specific population and context (COVID-19-HCWs) by the mental health specialists providing treatment, including: a) pharmacological prescription and follow-up, which was recorded as a procedure in approximately 40 % of the sessions for treating moderate to severe MHP; and b) two psychological techniques that seems to be essential tools to treat people suffering significant stressors outside their control (e.g., regular direct contact with COVID-19 patients that increases their risk of contagion):

1. Therapist’s “self-disclosure” of personal worries and feelings of vulnerability to COVID-19 as a health professional. By cautiously modeling openness and sharing intense feelings, therapists can use self-disclosure to enhance patients’ perception of their warmth and connection with them, and elicit more self-disclosure and positive responses on the part of the patient (Henretty and Levitt (2010), dispelling the widespread myth about the inherent difficulty of establishing a therapeutic alliance in non-face-to-face interventions (Berger, 2017).
2. The “use of metaphors,” specially to encourage patients to employe the techniques to manage maladaptive thoughts and emotions (see an example of a therapist-generated metaphor that can be part of a stock of metaphors for this in Table 4). The use of metaphors has demonstrated to be an effective conceptual and clinical strategy to facilitate therapeutic communication (Stine, 2005), information processing (Otto, 2000) and constructive change (Lenrow, 1966), and has been referred to as one of the most important therapeutic tools (Törneke, 2017) available to psychotherapists from different therapeutic orientations, including CBT.

Besides, a significant clinical improvement (as a measure of intervention’s effectiveness) was registered in all COVID-19-HCWs, and nearly 80% managed to improve after the intervention (even though more than 65% were moderately to severely ill at the beginning of the intervention). Additionally, a subset of patients who provided reports on acceptance were “totally satisfied” with the contents of the intervention and would recommend it to their colleagues (referred in implementation science as acceptability level) (Proctor et al., 2011).

In sum, our results indicate that the brief, remote, evidence-based intervention was a feasible and acceptable manner to attend emotional distress in Mexican COVID-19-HCWs, and 79.4% of participants demonstrated a significant improvement. These findings are congruent with previous reports on the effectivity of remote crisis intervention (Berrouiguet et al., 2018; Reinhardt et al., 2019) and the utility of helplines providing psychological first aid to first respondents during emergencies (Pekevski, 2013).

Other interesting results regards to COVID-19-HCWs’ help-seeking process during emotional crises and suicide risk, which might help to understand the type of vulnerable HCWs that could be attending helplines in future emergencies, as well as the efforts needed to increase the use of mental health services among other vulnerable subgroups of HCWs. First, most of those seeking help were women and COVID-19-HCWs associated with the medical profession. Gender differences may be due to the well-known greater tendency to seek and receive mental health care in women than men (Oliver et al., 2005) and the higher prevalence of anxious, depressive and stress-related MHP in women than men (Seedat et al., 2009); and differences between the type of HCWs could be explained given in Mexico (Robles et al., 2021a), as in other countries (Vizheh et al., 2020), those with a medical profession had higher frequencies of all MHP than psychologists, nurses and social workers.

Second, the main source of referral to the helpline was another HCW, which adds to evidence on the increased receipt of mental health care among healthcare professionals when it is suggested by someone in their social network (Dew et al., 1991), and the utility of informing key people—such as human resource colleagues and managers—about the signs of MHP and suicide risk and the services available to individuals requiring them) (World Health Organization, 2014). An example of an effective method to do so in general population that might be useful for this purpose in the future is the Mental Health First Aid (MHFA) Training and Research Program (for a description see: Kitchener and Jorm, 2008).

Third, the most important reason for COVID-19-HCWs to seek help and attend the intervention was testing positive for COVID-19, which has been reported as one of the main predictive factors of MHP among Mexican COVID-19-HCWs (Robles et al., 2021b). Along these same lines, one of the most frequently reported measures required to cope with COVID-19 by COVID-19-HCWs was biosafety equipment (Chen et al., 2020).

Fourth, according to the participating clinicians, a high proportion of COVID-19-HCWs seeking help presented some form of depression (with or without anxiety symptoms) and nearly a third part were at risk of suicide. This is in congruence with previous reports on mental health problems in COVID-19-HCWs. In Mexico, for example, according to Robles et al. (2021a,b) depression was one of the main common mental disorders among COVID-19-HCWs since the cluster of cases to the commentary transmission scenarios of the pandemic. Moreover, those with depression and alcohol abuse or dependence were at moderate suicide risk (vs. minimal or none), which highlight the need for monitoring of HCWs with this comorbidity.

This study has several and significant limitations. First, its hybrid effectiveness-implementation design (Curran et al., 2012) without a control group provides only preliminary evidence regarding the utility of the EBP implementation strategies and of the interventions themselves, which must be confirmed through controlled randomized clinical trials for better comparison and interpretation of their effects. Additionally, the absence of a follow-up evaluation prevents determining result’s maintenance in the long-term, and therefore is highly recommended in future studies on the field.

Another limitation is that, given the recruitment and sample selection method (of volunteer COVID-19-HCWs who engage in remote crisis intervention and/or suicide management), the results regarding MHP and suicide risk among COVID-19-HCWs should not be taken as estimates of prevalence or other epidemiological parameters. Moreover, an examination of whether the patterns of COVID-19-HCWs’ help-seeking during emotional crises and periods of suicide risk noted in this study apply across cultures and languages is warranted to make generalized conclusions in this regard.

Additionally, use of techniques and CGI ratings was reported by therapists rather that unbiased observers/raters, self-reported patients´ stress was based only on one item, and ratings of patients´ satisfaction with the intervention were asked directly by therapists, which might increase patients´ social desirability. Further assessments solving these limitations would improve the clarity of the effects of the intervention.

## Conclusion

The present study add evidence regarding the utility and acceptability of brief and remote crisis intervention and suicide risk management. Importantly, this evidence is produced among one highly vulnerable group in the context of a sanitarian emergency (COVID-19) and among inhabitants from middle-income countries, where scare information about the feasibility and effectivity of psychological interventions has been a constant (even more in the case of remote treatments). More women (than men), medicine undergraduate and graduate professionals (than other HCWs), moderately or more depressed seek this type of interventions, which substantially improve their mental health in nearly 80 % of the cases.

## Data availability statement

The datasets presented in this study can be found in online repositories. The names of the repository/repositories and accession number(s) can be found at: https://1drv.ms/f/s!AmLeJVltqurSgQJjOeerQliEDMHB?e=KOBYfk.

## Ethics statement

The study involved humans and was approved by Ramón de la Fuente Muñiz National Institute of Psychiatry Research Ethics Committee. The study was conducted in accordance with the local legislation and institutional requirements. The participants provided their written informed consent to participate in this study.

## Author contributions

RR: conceptualization, methodology, formal analysis, supervision, and writing – original draft. IS and FM: project administration, investigation, data curation, and writing – review & editing. AI, TE, R-DA, and MiE: investigation and writing – review & editing. FA: formal analysis, validation, and writing – review & editing. BC and ER: investigation, validation, and writing – review & editing. MAe: investigation, resources, and software. All authors contributed to the article and approved the submitted version.
